# Fear of hypoglycemia and its association with well-being, metabolic outcomes, and psychological health: A cross-sectional study in Danish adolescents with type 1 diabetes

**DOI:** 10.1371/journal.pone.0334243

**Published:** 2025-11-10

**Authors:** Amalie Hundahl, Louise Norman Jespersen, Stine Møller Sildorf, Emilie Bundgaard Lindkvist, Karl Bang Christensen, Steffen Ullitz Thorsen, Jannet Svensson

**Affiliations:** 1 Department of Paediatrics and Adolescent Medicine, Herlev University Hospital, Herlev, Denmark; 2 Department of Prevention, Health Promotion and Community Care, Copenhagen University Hospital—Steno Diabetes Center Copenhagen, Herlev, Denmark; 3 Department of Clinical and Translational Research, Copenhagen University Hospital—Steno Diabetes Center Copenhagen, Herlev, Denmark; 4 Section of Biostatistics, Department of Public Health, University of Copenhagen, Copenhagen, Denmark; 5 Department of Clinical Immunology, The Danish National University Hospital, Copenhagen, Denmark; 6 Department of Clinical Medicine, Faculty of Health and Medical Sciences, University of Copenhagen, Copenhagen, Denmark; Johns Hopkins: Johns Hopkins University, UNITED STATES OF AMERICA

## Abstract

**Introduction:**

Fear of hypoglycemia (FoH) among adolescents with type 1 diabetes (T1D) may hinder optimal diabetes treatment. This study aimed to investigate the association between FoH and metabolic outcome and psychological health.

**Materials and methods:**

Adolescents aged 11–18 with T1D and their parents were recruited from a diabetes clinic in Copenhagen from 2016–2017. Data were collected from 142 adolescents and 123 parents using Children’s Hypoglycemia Index (CHI), the World Health Organization-Five Well-Being Index, the Adherence in Diabetes Questionnaire, and DISABKIDS (health-related quality of life). Associations between CHI scores and clinical, demographic, and psychological data were analyzed. Rasch modeling was used for psychometric validation of Danish version of CHI.

**Results:**

Higher CHI scores were observed in females (p = 0.01). Adolescent and parental CHI scores were significantly correlated across CHI subscales (r = 0.20–0.33). With increasing CHI scores, we observed a decrease in well-being (p = 0.004), and health-related quality of life (p=<0.0001–0.007). No associations were found with HbA1c, treatment methods, blood glucose monitoring, diabetes duration, or adherence (p = 0.06). The Danish version of the CHI proved valid and useful for detecting FoH. We found an acceptable fit to the Rasch model across all three subscales.

**Conclusion:**

Though not related directly to treatment outcome FoH might be an important indicator of psychological health in adolescents with T1D.

## Introduction

Individuals with type 1 diabetes (T1D) constantly face the challenge of maintaining a stable level of blood glucose. Hypoglycemia occurs if too much insulin is administered relative to the turnover of carbohydrates. The occurrence of severe hypoglycemia have dropped over the past decade from 3 to 1.7 events per 100 person years, parallel with improved glycemic outcome [1]. Although modern diabetes treatments have reduced the overall incidence of severe hypoglycemia – such as, episodes of unconsciousness, convulsion and the unpleasant feeling of low blood glucose, this can still be frightening and even traumatic, potentially contributing to fear of hypoglycemia (FoH), and thereby affecting diabetes [2]. In 2005, the Children’s Hypoglycemia Index (CHI) was introduced by Kamps et al. [3]. CHI was designed to quantify FoH in families with children aged 8–16 with T1D. The CHI questionnaire exists in a childhood and parent versions, each measuring FoH. However, these instruments have not previously been validated in a Danish population.

Research has confirmed that experiencing severe hypoglycemia increases FoH in both adults, adolescents, and parents [2,4,5]. In children and adolescents FoH is linked to impaired mental health, reduced health-related quality of life (QoL), and higher anxiety levels [2,4–7]. A recent prospective study of parental FoH found that parents’ worry increased within the first year following their child’s diabetes diagnosis and was associated with the child spending less time in the target glucose range [8]. Other studies have found that children with T1D were less physically active than their peers without T1D and that FoH was often stated as one of the major barriers for physical activity [9], however, this is questioned by recent research [10,11]. FoH is also associated with greater glycemic variability and higher calorie intake in adults [12], whereas the association between FoH and HbA1c values is inconsistent in both adults and children [2,4]. Although insulin pump therapy is linked to lower risk of severe hypoglycemia [13], the impact of new diabetes device technology on FoH is less clear [14,15]. FoH may increase the frequency of blood glucose monitoring, but continuous glucose monitoring did not stop parents from worrying about hypoglycemia [4,14]. Yet automated insulin delivery systems may reduce FoH among parents [15]. Parental FoH does not appear to be strongly correlated with adolescent FoH, as parents report significantly higher mean scores than adolescents. Moreover, while FoH increases with the child’s age among adolescents, it tends to decrease among parents [16], demonstrating the importance of investigating FoH in both adolescents and parents. A systematic review from 2021, concluded that there is insufficient evidence regarding the impact of hypoglycemia on quality of life (QoL) in children and adolescents with T1D [17] and that there is a need for further research to examine this relationship, ideally using hypoglycemia-specific QoL measures [17]. Thus, our study aimed to investigate the association between adolescents’ FoH and metabolic outcome and psychological health as well as association to parental FoH.

## Materials and methods

### The study population and questionnaire measurements

Adolescents with T1D aged 11–18 years were approached during their regular clinical visits at the pediatric outpatient clinic of Northwestern Copenhagen from 1. October 2016–31. December 2017. They were asked to participate in a larger questionnaire survey covering diabetes psychosocial challenges.

The questionnaires were send to the families by e-mail included the CHI (parent and child version), the World Health Organization-Five Well-Being Index (WHO-5) assessing psychological well-being [18], the DISABKIDS questionnaire with six general and two diabetes-specific subscales measuring health-related quality of life for children with chronic medical diseases, and the Adherence in Diabetes Questionnaire (ADQ) evaluating self-reported adherence [19]. Parents were asked to complete only the parent version of the CHI, while adolescents were asked to complete all questionnaires. Reminders were sent twice to non-responders. Demographic, clinical, and metabolic data were collected retrospectively from medical records and linked to the time the questionnaires were completed.

For this study, the original English version of CHI [3] was translated into Danish by two Danish pediatric researchers. Any discrepancies were discussed until a consensus was reached. The Danish CHI was then backward translated by a native English speaker, fluent in Danish and approved by the author of the original CHI questionnaires, J. Kamps. CHI consists of 24 questions separated into three subscales: Eight questions addressed general concerns related to living with diabetes (*General fear* subscale), seven assessed situational fear (*Situation* subscale), and nine focused on behaviors to avoid hypoglycemia (*Behavior* subscale). The responses range from 1–5 on a 5-point Likert scale with higher scores equivalent to greater fear.

### Statistical approach

To investigate the relationship between CHI scores, clinical metrics, and other questionnaire responses, total CHI scores were categorized into three groups; low (≤ Q1), median (> Q1 and < Q3), and high (≥ Q3), based on quartile divisions for the 142 adolescents. The Kruskal–Wallis test was used to compare the distribution of continuous variables across CHI tertiles. To explore differences between specific groups (Q1, Q2 + Q3, Q4), we applied the Dwass–Steel–Critchlow–Fligner (DSCF) method, which includes adjustment for multiple testing. For binary variables, the chi-square test of independence was used. Spearman’s rank correlation was employed to assess correlations between childhood and parental CHI subscale scores. A significance level of 0.05 was used for all statistical tests including those adjusted for multiple comparisons.

To validate the Danish version of CHI, an overall fit to the Rasch model was assessed using the Andersen conditional likelihood ratio test [20], and individual item fit was evaluated using the conditional outfit test statistic [21]. We also evaluated item fit graphically using conditional item characteristic curves [22]. We tested for local response dependence (LRD) using two partial gamma coefficients for each item pair [23], and differential item function (DIF) [24] for sex and age groups using log-linear Rasch model tests [25] and item screening [26]. Where evidence of LRD was found, we also evaluated item fit using graphical Rasch models [26]. In all analyses, we adjusted p-values using the Benjamini-Hochberg [27] procedure to control the false discovery rate (FDR). For items where misfit was significant after adjustment for multiple testing, we also evaluated item fit graphically using conditional item characteristic curves [23]. Furthermore, the alignment between item locations and person locations was visualized. Analyses were done using DIGRAM [28] and R [29]. No formal sample size calculation was performed; however, there was an *a priori* goal of obtaining more than 100 respondents.

### Ethics approval

The study was approved by the Danish Data Protection Board and Patient Safety Authority with ID HGH-2016-029 04471. In Denmark research including questionnaire data and clinical information from health journals and no biological samples do not need ethical approval. All included patients and parents provided written informed consent prior to study inclusion.

## Results

**Fig 1:**
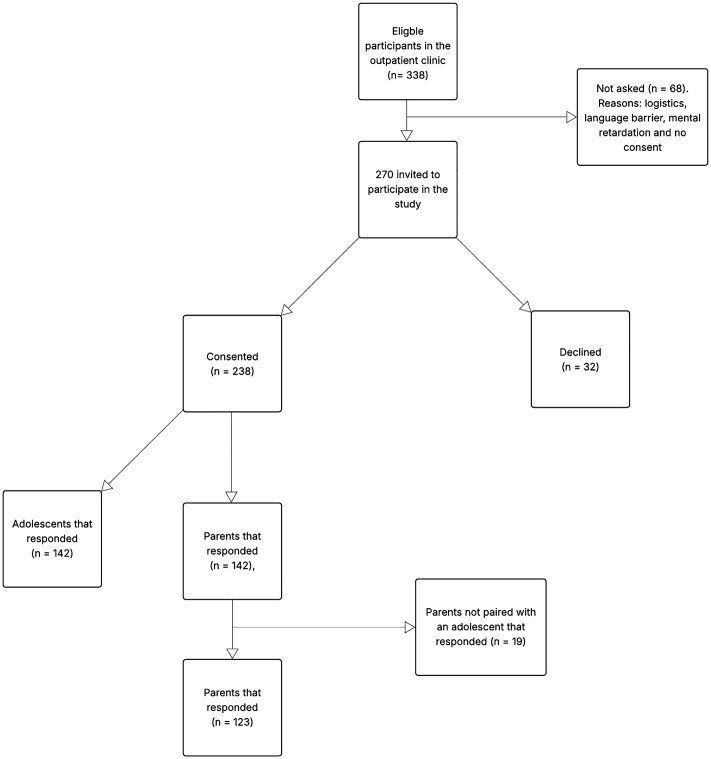
Participant flowchart.

Of 338 eligible patients with T1D at the outpatient clinic, 270 were approached for inclusion (68 were not approached due to logistical reasons, language barriers, cognitive impairment, or lack of consent). Among those approached, 238 agreed to participate and 32 declined. In total, 142 adolescents completed all questionnaires, and 123 parents responded to the childhood or parent version of the CHI survey (Fig 1).

The 142 participants had a mean age of 14.9 years (SD 2.1); 78 were female. Overall, the mean diabetes duration was 5.4 years (SD 3.3), and the mean HbA1c was 63.3 mmol/mol (SD 14.3).

### CHI score in relation to demographic, metabolic, and psychometric data

Main results are presented in Table 1—we encourage readers to evaluate the p-values up against the demographic, metabolic, and psychometric trends over the low (<Q1), median (Q1-Q3) and high (>Q3) CHI score groups.

**Table 1:** Total childhood CHI score and demographic, metabolic and questionnaire data.

		Low group CHI ≤Q1 (N = 36)	Median group CHI > Q1 and <Q3 (N = 71)	High group CHI ≥ Q3 (N = 35)	P-value	P-value DSCF
**Demographic and metabolic data**						
Gender	Male, N (%)	24 (37.5%)	28 (43.8%)	12 (18.8%)	**0.009**	
	Female, N (%)	12 (15.4%)	43 (55.1%)	23 (29.5%)		
Treatment method	Pump users, N (%)	21 (25.0%)	43 (51.2%)	20 (23.8%)	0.94	
	Pen users, N (%)	15 (25.9%)	28 (48.3%)	15 (25.9%)		
Age (years), Median (range)		14 (11-18)	15 (11-18)	15 (11-18)	0.07	
HbA1c (mmol/mol), Median (range)		60 (38-105)	60 (33-108)	64 (41-89)	0.81	
Diabetes duration (years), Median (range)		4.61 (0.85-14.1)	5.46 (0.27-13.4)	4.97 (0.0-14.0)	0.63	
Daily frequency of blood glucose checks, Median (range)		5.25 (0-14)	5.4 (1.2-30)	4.6 (0.5-10)	0.55	
**Questionnaire data scores (median (min-max))**						
WHO-5		74 (24-100)	70 (30-100)	60 (26-94)	**0.007**	**Q1:M 0.24** **Q1:Q4 0.007** **M:Q4 0.09**
ADQ		3.88 (2.68-5.00)	3.70 (2.21-4.89)	3.58 (2.40-5.00)	0.06	
DISABKIDS: Mental Independence		26 (16-30)	26 (18-30)	23 (13-30)*	**0.003**	**Q1:M 0.85** **Q1:Q4 0.009** **M:Q4 0.005**
DISABKIDS: Physical Limitations		24 (15-30)	24 (16-30)	20 (14-29)	**<0.0001**	**Q1:M 0.61** **Q1:Q4 < 0.001** **M:Q4 < 0.001**
DISABKIDS: Mental Emotions		30 (19-35)	28 (15-35)	23 (7-33)	**<0.0001**	**Q1:M 0.08** **Q1:Q4 < 0.001** **M:Q4 0.005**
Social Exclusion		27 (17-30)	26 (14-30)	23 (16-30)	**0.002**	**Q1:M 0.38** **Q1:Q4 0.003** **M:Q4 0.014**
Social Inclusion		25 (13-30)	25 (15-30)	23 (14-29)	**0.0008**	**Q1:M 1.00** **Q1:Q4 0.004** **M:Q4 0.002**
Physical Treatment		24 (9-30)	23 (9-30)	16 (6-27)	**<0.0001**	**Q1:M 0.29** **Q1:Q4 < 0.001** **M:Q4 < 0.001**
Diabetes Impact		23 (11-30)	21 (12-30)	18 (11-27)	**<0.0001**	**Q1:M 0.02** **Q1:Q4 < 0.001** **M:Q4 < 0.001**
Diabetes Treatment		15 (5-20)	13 (4-20)	9 (4-16)	**<0.0001**	**Q1:M 0.04** **Q1:Q4 < 0.001** **M:Q4 < 0.001**
Parental CHI, N = 123		N = 32, 32 (22–57)	N = 65, 37 (21–63)	N = 26, 41.5 (26–57)	0.004	**Q1:M 0.15** **Q1:Q4 0.07** **M:Q4 0.004**

**Legend**: CHI, Children’s Hypoglycemia Index; DSCF, Dwass–Steel–Critchlow–Fligner method; WHO-5; the World Health Organization-Five Well-Being Index; ADQ; Adherence in Diabetes Questionnaire.

Females were more likely to be in the high CHI group than males (p < 0.01). On the contrary there were no signs of different distribution of age, insulin delivery method, daily frequency of blood glucose measurements, HbA1c, or diabetes duration among the low, medium, and high CHI groups. Because no participants were hospitalized with ketoacidosis and only two experienced severe hypoglycemia, a potential association with CHI score could not be assessed. With increasing overall CHI scores, there was no statistically significant decrease in ADQ score (p = 0.06), while significant decreases were observed in WHO-5 score (p = 0.007) and DISABKIDS subscale scores (p < 0.0001–0.003) (Table 1). For WHO-5, only the upper 25% differed significantly from the lower 25%. In the DISABKIDS subscales, the upper 25% differed significantly from the median and lower 25%. Only the subscales *Diabetes impact* and *Diabetes treatment* showed significant differences across all three groups after adjustment for multiple testing (Table 1).

### Correlation between childhood and parental fear of hypoglycemia

There was an increasing trend in parental CHI when grouped by adolescent CHI categories (Table 1). Indicating some agreement of worries in the family in these groups. Spearman’s correlation revealed a weak positive association between adolescent and parental CHI scores across all three subscales, ranging from r = 0.20 for the *Behavior* subscale (p = 0.02) to r = 0.29 for the *Situation* and r = 0.31 for the *General fear* subscales, with all p-values < 0.01.

### Validity of the Danish Childhood Version of CHI

The overall model fit for each subscale, presented in Table 2, showed some significant age and sex differences.

**Table 2:** Model fit for childhood CHI subscales.

Subscale	Variable	χ²	df	P-value	FDR
Situation	Score groups	21.0	25	0.6938	0.6938
	Girl	33.8	25	0.1113	0.1670
	Over 14	38.8	25	**0.0390**	0.1170
General fear	Score groups	25.8	29	0.6373	0.6373
	Girl	44.2	29	**0.0351**	0.1053
	Over 14	26.6	29	0.5909	0.6373
Behavior	Score groups	28.7	28	0.4275	0.4275
	Girl	34.2	28	0.1956	0.2934
	Over 14	42.2	28	**0.0413**	0.1239

**Legend:** df: degrees of freedom FDR: False Discovery Rate.

After adjusting for multiple testing, age and sex differences in total subscale scores were no longer significant. Item fit statistics, shown in Table 3, also showed no significant differences indicated a good fit.

**Table 3:** Evaluation of item fit, differential item function (DIF) and local response dependence (LRD) in the initial Rasch analyses.

Subscale	Item fit				DIF							
					With respect to sex				With respect to age group			
	Item	OUTFIT	P	FDR	CLR	DF	P	FDR	CLR	DF	P	FDR
Situation	1	0.58	0.3792	0.6637	2.1	3	0.5431	0.5858	2.1	3	0.5440	0.5858
	2	1.27	0.0260	0.0909	6.2	4	0.1860	0.4788	5.6	4	0.2280	0.4788
	**3**	**0.53**	**0.0059**	**0.0411**	6.3	4	0.1807	0.4788	9.8	4	0.0444	0.3108
	4	0.81	0.1852	0.4322	3.8	4	0.4387	0.5858	5	4	0.2838	0.4967
	5	1.04	0.7115	0.8300	3.6	4	0.4641	0.5858	10.3	4	0.0355	0.3108
	6	0.91	0.8422	0.8422	4.2	3	0.2394	0.4788	4.4	3	0.2168	0.4788
	7	0.90	0.5532	0.7745	4.1	4	0.3904	0.5858	2.8	4	0.5908	0.5908
General fear	8	1.04	0.7995	0.7995	7.5	4	0.1125	0.3725	5	4	0.2880	0.5760
	9	0.91	0.5016	0.7995	3.2	4	0.5226	0.7765	1.7	4	0.7954	0.9044
	10	0.95	0.7802	0.7995	3.2	4	0.5321	0.7765	6.9	4	0.1397	0.3725
	11	0.71	0.0225	0.0899	5.7	4	0.2246	0.5134	1.4	4	0.8479	0.9044
	12	1.10	0.3790	0.7581	7.4	4	0.1165	0.3725	8	4	0.0913	0.3725
	13*	**1.61**	**0.0004**	**0.0034**	2.6	4	0.6309	0.7765	2.6	4	0.6205	0.7765
	14	0.79	0.3739	0.7581	7.2	3	0.0665	0.3725	0.3	3	0.9691	0.9691
	15	1.13	0.6388	0.7995	7.7	3	0.0531	0.3725	2.1	3	0.5565	0.7765
Behavior	16	0.92	0.5635	0.7246	3	3	0.3960	0.5091	1.7	3	0.6311	0.7050
	17	1.18	0.1334	0.3002	6.1	4	0.1941	0.5091	4.4	4	0.3506	0.5091
	18	0.97	0.9470	0.9470	4.8	3	0.1833	0.5091	7.9	3	0.0489	0.5091
	19	0.75	0.0956	0.2868	7.2	3	0.0673	0.5091	3.1	3	0.3773	0.5091
	20	0.59	0.0177	0.0797	1.8	3	0.6148	0.7050	3.1	3	0.3824	0.5091
	21*	**1.41**	**0.0008**	**0.0075**	4.5	4	0.3418	0.5091	5.8	4	0.2121	0.5091
	22	0.66	0.3676	0.5514	1.6	3	0.6658	0.7050	4.5	3	0.2091	0.5091
	23	0.95	0.8434	0.9470	3.1	3	0.3763	0.5091	0.6	3	0.9036	0.9036
	24	1.22	0.2100	0.3779	3	3	0.3948	0.5091	4.1	3	0.2487	0.5091

**Legend:** Item 13 and 21 do not fit the Rasch model.

Evidence of LRD in all subscales (items 3 and 4; items 9 and 11; items 19 and 20). For each of these item pairs one of the items should be excluded. (For detailed list of all items see supplemental materials).

The analysis revealed evidence of LRD for the item pair (3, 4) the gamma-values were 0.54 (95% CI: 0.10 to 0.97) and 0.53 (95% CI: 0.10 to 0.95), respectively. This means the response to item 3 is not significantly different from item 4 and therefore they contribute with less information than two separated items. These items concern FoH when with friends or in school, respectively. The LRD indicates one of these items can be omitted. After adjustment for multiple testing there was no evidence of DIF (all FDR values above 0.05; Table 3) when item with no fit and LRD was removed. A graphical Rasch model taking LRD into account had a perfect fit.

**Fig 2:**
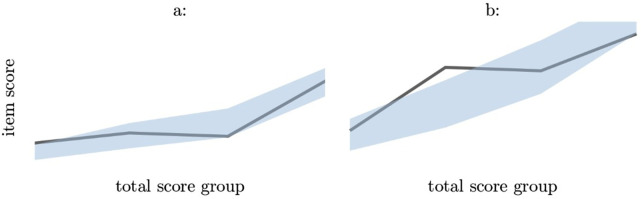
Graphical evaluation of item misfit using conditional item characteristic curves. The horizontal axis shows the total scale score divided into four groups, while the vertical axis shows the item score. Grey line indicates observed data, while the shaded area indicates expected values.

For the *General Fear* subscale, the overall model fit was acceptable, but item 13 did not fit the Rasch model well as shown in Table 3 and Fig 2a.

Significant evidence of LRD was found for the item pair (9, 11) where the gamma-values were 0.95 (95% CI: 0.87 to 1.00) and 0.96 (95% CI: 0.88 to 1.00), respectively. These items are related to fear of experiencing seizures or attacks due to hypoglycemia and one may subsequently be omitted. There was no evidence of DIF in this subscale. A graphical Rasch model taking LRD into account showed a better fit to the data.

For the *Behavior* subscale, the overall model fit was excellent, but item 21 did not fit the Rasch model well Table 3 and Fig 2b. There was significant evidence of LRD between item 19 and item 20. For the item pair (19, 20) the gamma-values were 0.77 (95% CI: 0.55 to 0.99) and 0.80 (95% CI: 0.63 to 0.98), respectively. Both items were regarding keeping blood glucose high, the LRD means one can be omitted. There was no evidence of DIF in the *Behavior* subscale. A graphical Rasch model taking LRD into account showed a better fit to the data.

In summary, the Rasch analyses confirmed the validity of the CHI while also disclosing three item pairs with LRD. Visualization of the person locations and the item locations indicates good alignment between the location of the persons and the location of the items (Supplementary Materials, S1 Fig). Cronbach’s alpha was above 0.90 for all items before removing items 4, 11, 13, 20, and 21, and remained above 0.88 for all items after their removal.

The targeting is presented in Supplementary Materials, S1 Fig. It seems appropriate as there is an overlap of respondents and item location.

### Sensitivity analyses

A sensitivity analysis was performed excluding the two items that did not initially fit the Rasch model (items 13 and 21) and the three items that were deemed redundant based on the evidence of LRD (items 4, 11, and 20). This yielded similar results to the original regarding associations with parental CHI score, the WHO-5, ADQ, and DISABKIDS subscales. The higher score in females also applied when CHI was divided into subscales and when non-fitting/locally dependent items were omitted.

The original English and final translated version of CHI are presented in the Supplemental materials, S1 Table.

## Discussion

Our study shows that increasing CHI score was significantly associated with decreased well-being, health-related quality of life, but not adherence. Metabolic parameters including HbA1c were not associated with CHI score. The Danish version of CHI was overall valid and reliable. Further, the subscales were reasonably well-targeted to the population.

As in some previous studies, we found higher FoH scores among females, although the impact of sex is not consistently reported in the literature [4,7,16,30]. In two cross-sectional study of 187 adolescents in Saudi Arabia and 96 Dutch adolescents, female sex was associated with higher FoH scores [7,30]. In a small cross-sectional study (N = 39) of adolescents, no sex differences in FoH scores were detected [2], nor were they reported in the referenced review [4]. In many questionnaires concerning mental health females tend to have higher scores than males [31]. However, in our study we found no statistical evidence of difference item interpretation between males and females, this implies girls do have higher FoH and we can use same cut-off for FoH in boys and girls. We have previously demonstrated signs of earlier transfer of responsibility from parents to girls [32]. The higher CHI score among females may reflect personal traits but could also represent girls being left with too much responsibility. Within families, we found a weak positive correlation between adolescent and parental subscale CHI scores, which could be explained by both heritable personality traits and environmental factors. The lack of high agreement between parent and adolescent FoH is in accordance with other studies [2,4].

Although the risk of hypoglycemia decreases with advanced diabetes technologies, [13,33,34] we were unable to show any association between FoH and type of insulin administration (automated insulin delivery systems were not available when our study was conducted). The lack of association to treatment could be due to the free access to technology in Denmark, where anyone with anxiety of needles or FoH would be offered insulin pump and sensor. We did not find a significant negative association between self-reported adherence and CHI score, only a trend. Patients with high FoH may be more uncomfortable dealing with low blood glucose leading to avoidance coping, reported as low adherence [19,35]. In a previous prospective cohort study of Danish children and adolescents 10−17 years of age with T1D better adherence was associated with lower levels of HbA1c in the following 11 years [35]. Still, in our study FoH was not associated with HbA1c levels, perhaps reflecting the cross-sectional design, which do not capture behavioral changes. Lastly, we found a negative association between CHI scores and both WHO-5 and DISABKIDS scores indicating lower well-being and poorer health-related quality of life, respectively in accordance with the Dutch study [7]. A recent review including 27 papers found insufficient evidence for the association between FoH and decreased health-related quality of life in children [17]. The main problem was heterogenous definitions and outcomes as well as different definitions of hypoglycemia as well as fear of hypoglycemia. An association between parental FoH and QoL have been documented [16]. It is not clear from our cross-sectional study whether FoH is a stressful, mental burden that worsens mental health and reduces quality of life. Or if some children may be more vulnerable and prone to report lower quality of life and higher FoH. To investigate this, longitudinal prospective studies are needed.

The strength of this study lies in its use of a previously validated questionnaire, which was translated and adapted for our context. Additionally, the low rate of missing data among the 142 included adolescents, including responses to additional questionnaires, improving the reliability of our findings. However, there are some limitations, only 60% of those who initially agreed to participate completed the questionnaire. This low response rate was likely influenced by insufficient reminders and the survey load, including multiple time-consuming questionnaires. The sample may therefore not be representative of the population increasing the risk of selection bias. The cross-sectional design also does not allow for causal interpretations.

### Implications for research and practice

Future studies should aim to unveil the importance of FoH when using advanced diabetes technologies and follow FoH over time to identify a cut-off value detecting problematic FoH. Such studies could assist clinicians in improving treatment adherence, mental well-being, and subsequently quality of life. When administering the original CHI questionnaire, practitioners should be aware that certain subscales contain locally dependent items, suggesting that these questions, along with those not conforming to Rasch analysis, could be excluded. Further, it is important that the questionnaires are adapted to address the evolving treatment approaches. A shorter version of CHI would, in our opinion, be beneficial due to shorter completion time.

## Conclusions

The association between FoH and mental well-being highlights the relevance of addressing hypoglycemic fear in adolescents. Since parental FoH was only weakly correlated to adolescent FoH, it is important to investigate FoH in both adolescents and parents. Our results confirm that CHI is a valid tool for assessment of hypoglycemia-related fear and behavior among adolescents with T1D.Validations in this Danish population show that five of the CHI items should be excluded without hampering the validity.

## Supporting information

S1 Fig Evaluation of the alignment between person locations and item locations. The horizontal axis is the latent continuum, while the vertical black line indicates the average value of the respondents. Blue bars below the horizontal axis shows the location of the items, while blue bars above are a histogram of the person locations.
(TIF)

S1 Table Danish translation and English version of childhood CHI. (DOCX)

## Abbreviations

T1D: Type 1 Diabetes
FoH: Fear of Hypoglycemia
CHI: Children’s Hypoglycemia Index
QoL: Quality of Life
WHO-5: The World Health Organization-Five Well-Being Index
ADQ: Adherence in Diabetes Questionnaire
LDR: Local Response Dependence
DIF: Differential Item Function.
