# Supplementary figures and images for: Fear of hypoglycemia and its association with well-being, metabolic outcomes, and psychological health: A cross-sectional study in Danish adolescents with type 1 diabetes
Source: PLoS One. 2025 Nov 10;20(11):e0334243. doi: 10.1371/journal.pone.0334243 (PMC12599912; doi:10.1371/journal.pone.0334243)

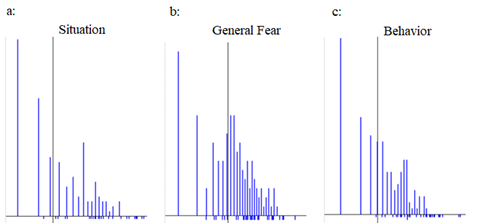

Supplement: S1 Fig — The horizontal axis is the latent continuum, while the vertical black line indicates the average value of the respondents. Blue bars below the horizontal axis shows the location of the items, while blue bars above are a histogram of the person locations. (TIF) [file pone.0334243.s001.tif]
