# Supplementary material for: Fear of hypoglycemia and its association with well-being, metabolic outcomes, and psychological health: A cross-sectional study in Danish adolescents with type 1 diabetes
Source: PLoS One. 2025 Nov 10;20(11):e0334243. doi: 10.1371/journal.pone.0334243 (PMC12599912; doi:10.1371/journal.pone.0334243)
Supplement: S1 Table — (DOCX) [file pone.0334243.s002.docx]

| **Item number:** | **Danish translation:** | **English:** |
| --- | --- | --- |
| **Answer options item 1-15:** | **1=Ikke bange**  **2=Lidt bange**  **3=Bange**  **4=Meget bange**  **5=Ekstremt bange** | **1=Not Afraid**  **2=A Little Afraid**  **3=Afraid**  **4=Very Afraid**  **5=Extremely Afraid** |
| 1 | At have lavt blodsukker, når jeg er hjemme hos mine forældre, gør mig… | Having low blood sugar when I am home with my parents makes me feel… |
| 2 | At have lavt blodsukker, mens jeg sover om natten, gør mig… | Having low blood sugar when I am asleep at night makes me feel… |
| 3 | At have lavt blodsukker i skolen gør mig… | Having low blood sugar when I am at school makes me feel… |
| 4 | At have lavt blodsukker, når jeg er ude med venner, gør mig… | Having low blood sugar when I am out with a group of friends makes me feel… |
| 5 | At have lavt blodsukker, når jeg er alene, gør mig… | Having low blood sugar when I am alone makes me feel… |
| 6 | At have lavt blodsukker, når jeg er på diabetes-sommerlejr, gør mig… | Having low blood sugar when I am at diabetes summer camp makes me feel… |
| 7 | At have lavt blodsukker, når jeg er på lejrskole med børn uden diabetes, gør mig… | Having low blood sugar when I am at a camp that is not for kids with diabetes makes me feel… |
| 8 | At opføre mig fjollet, når mit blodsukker bliver lavt, gør mig… | Acting silly when my blood sugar gets low makes me feel… |
| 9 | At falde om på grund af lavt blodsukker gør mig … | Passing out from low blood sugar makes me feel… |
| 10 | At være alene, når mit blodsukker er lavt, og der ikke er nogen, der kan hjælpe mig, gør mig… | Being alone when my blood sugar is low and having no one to help me makes me feel… |
| 11 | At få krampeanfald, når mit blodsukker er lavt, gør mig… | Having a seizure when my blood sugar is low makes me feel… |
| 12 | Ikke at kunne mærke når mit blodsukker er lavt, gør mig… | Not realizing when my blood sugar gets low makes me feel… |
| 13 | At lave en fejl i skolen fordi mit blodsukker er lavt, gør mig… | Making a mistake at school because my blood sugar is low makes me feel… |
| 14 | At gøre mig selv til grin, fordi mit blodsukker er lavt, gør mig… | Embarrassing myself when my blood sugar is low makes me feel… |
| 15 | At bede om hjælp, når mit blodsukker er lavt, gør mig… | Asking for help when my blood sugar is low makes me feel… |
| **Answer options item 16-24:** | **1=Aldrig**  **2=Næsten aldrig**  **3=Nogle gange**  **4=For det meste**  **5=Hele tiden** | **1=Never**  **2=Hardly ever**  **3=Sometimes**  **4=Most of the Time**  **5=All of the Time** |
| 16 | Jeg bekymrer mig om at få lavt blodsukker, selv når mit blodsukker-niveau er ok | I worry about having low blood sugars even when my blood sugar range has been ok. |
| 17 | Jeg holder mit blodsukker højt, når jeg er sammen med mine venner, så jeg ikke behøver at bekymre mig om lavt blodsukker | I keep my blood sugar high when I am with friends so I won’t have to worry about it going low. |
| 18 | Jeg kan ikke lide at være alene, fordi jeg er bekymret for at få lavt blodsukker | I don’t like to be alone because I am worried that my blood sugar might get low. |
| 19 | Jeg tager mindre insulin, end jeg burde, fordi jeg ikke vil have lavt blodsukker | I take less insulin than I should because I don’t want my blood sugar to get low. |
| 20 | Jeg holder mit blodsukker højere, end det burde være, så jeg ikke behøver bekymre mig om lavt blodsukker | I keep my blood sugar higher than it should be so I won’t have to worry about it getting low. |
| 21 | Jeg tjekker ofte mit blodsukker, fordi jeg er bange for, at det skal blive lavt | I check my blood sugar often because I am afraid it might get low. |
| 22 | Jeg vil gerne til lægen oftere end nu, fordi jeg er bekymret for, at mit blodsukker skal blive for lavt | I want to go to the doctor more often than I do now because I am worried about my blood sugar being low. |
| 23 | Jeg spiser mere mad, end jeg burde, for at undgå at få lavt blodsukker | I eat more food than I should because I don’t want my blood sugar to get low. |
| 24 | Jeg spiser et større mellemmåltid før sengetid, end jeg burde, så jeg er sikker på, at mit blodsukker ikke bliver for lavt midt om natten | I eat a bigger snack at bedtime than I should so I can be sure my blood sugar won’t get low in the middle of the night. |

Items marked in gray can be omitted as they are redundant due to local response dependence (LRD) or did not fit the overall RASCH model (item 13+21).
